# High Glucose Decreases Expression and Activity of p-glycoprotein in Cultured Human Retinal Pigment Epithelium Possibly through iNOS Induction

**DOI:** 10.1371/journal.pone.0031631

**Published:** 2012-02-17

**Authors:** Yuehong Zhang, Chunmei Li, Xuerong Sun, Xielan Kuang, Xiangcai Ruan

**Affiliations:** 1 Departments of Ophthalmology, and Anesthesiology, First Municipal People's Hospital of Guangzhou, Affiliated Hospital of Guangzhou Medical College, Guangzhou, China; 2 Zhongshan Ophthalmic Center, Sun Yat-sen University, Guangzhou, China; Children's Hospital Boston/Harvard Medical School, United States of America

## Abstract

Inhibition of p-glycoprotein under hyperglycemic conditions has been reported in various barrier tissues including blood-brain barrier, intestine, and kidney, and has been linked to significant clinical complications. However, whether this is also true for the outer blood-retinal barrier constituted by retinal pigment epithelium, or has a role in pathogenesis of diabetic retinopathy is not yet clear. In this study, using cultured human retinal pigment epithelium cell line D407, we found that high glucose exposure induced a significant decrease in p-glycoprotein expression both at mRNA and at protein levels, accompanied by an attenuated p-glycoprotein activity determined by intracellular rhodamine 123 retention. In marked contrast, the expressions of both mRNA and protein levels of inducible nitrate oxide synthase (iNOS) increased, and were accompanied by increased extracellular nitrate/nitrite production by Griess reaction. In addition, mRNA levels of nuclear receptors revealed a decreased expression of pregnane X receptor after the exposure of high glucose. However, the subsequent alterations in production of nitrate/nitrite, functional expression of p-glycoprotein, and mRNA levels of pregnane X receptor were partially blocked when pretreated with S,S′-1,3-phenylene-bis(1,2-ethanediyl)-bis-isothiourea•2HBr (PBITU), a selective iNOS inhibitor. Moreover, the effects of PBITU were antagonized with the addition of L-arginine, a substrate for NO synthesis. Our *in vitro* results suggest for the first time that iNOS induction plays a novel role in decreased p-glycoprotein expression and transport function at the human outer blood-retinal barrier under hyperglycemic conditions and further support the concept of inhibiting iNOS pathway as a therapeutic strategy for diabetic retinopathy.

## Introduction

The retinal pigment epithelium (RPE) is a monolayer of pigmented cells located between the neural retina and the choroid, and therefore constitutes the outer blood-retinal barrier (BRB, for a review see ref. 3). The inner BRB is mainly constituted by endothelial cells. Because the RPE forms the outer BRB and is essential for removal of waste products and entry of nutrients into the retina, any disturbance in normal transport function of these cells necessarily has detrimental consequences for the retina. It is well documented that defects in RPE function may underlie a number of sight-threatening conditions, such as age-related macular degeneration, proliferative vitreoretinopathy, and diabetic retinopathy (DR) [1], [2], [3].

Diabetic retinopathy is the most serious complication of diabetic eye disease and one of the most common leading causes of irreversible blindness worldwide [4], [5]. The role of hyperglycemia in the development of DR has now been strongly affirmed. Breakdown of the retinal barrier function is considered to be the basis of the pathogenesis of DR [3], [6]. Numerous studies on pathogenesis of DR have been focused on the impairment of the neural retina and the inner BRB [4], [7]. It is increasingly recognized that impairment of the outer BRB plays an important role in the initiation and progress of early DR [3], [8]. We thus suspected that the abnormal elevated blood glucose levels during the development of DR may potentially disturb the barrier function at the outer BRB. Indeed, Liu and colleague [9] have reported that the alterations in expression of efflux pumps such as p-glycoprotein (P-gp) could lead to damages in barrier integrity in diabetes and could be restored by insulin therapy. However, the direct data for the effects of high glucose or hyperglycemia on the transport functions at the outer BRB are not yet available.

As a best-characterized efflux transport protein, P-gp is considered by far the most important among efflux transporters expressed in mammalian tissues [10]. P-gp is a membrane phosphoglycoprotein encoded by the multidrug resistance MDR1 gene which has ATP-dependent drug efflux pump function [10]. Besides the overexpression of P-gp in multidrug-resistant cell lines, it is mainly expressed in cells with active secretory and excretory functions, such as renal proximal tubule, intestinal epithelium and blood-brain barrier [10], [11], [12], [13], which indicates the primary role of P-gp in secretion and transport. Recently, basal P-gp functional expression was also detected in the human RPE cells [14], [15]. Attenuated P-gp expression and functions by high glucose have been reported in various tissues, such as blood-brain barrier [16], [17], intestine [11], liver [18], [19], and kidney [19], [20], and are linked to significant clinic complications. However, whether this is also true for the outer BRB or has a role in pathogenesis of DR is still mysterious. In the present study, we show that inducible nitrate oxide synthase (iNOS) signaling pathway is induced in the cultured human RPE cells by the exposure of high glucose, and high glucose-induced iNOS pathway results in the inhibition of functional expression of P-gp and transcriptional expression of pregnane X receptor (PXR), a nuclear receptor that regulates expression of drug metabolizing enzymes and efflux transporters [13], [21]. We propose that iNOS induction by high glucose suppress the transcription of PXR, and thereby inhibit the expression and activity of P-gp at the human outer BRB.

## Materials and Methods

### Cell Culture

The human RPE cell line D407 was generously given by Richard Hunt (Department of Immunology and Pathology, University of South Carolina Medical School, Columbia, SC, USA). These cells were shown to possess most metabolic and morphologic characteristics of RPE cells in vivo [22] and express functional P-gp [23]. The D407 cells were incubated in DMEM with normal (5.5 mM), intermediate (12.5 mM) or high (25 and 50 mM) glucose, supplemented with 10% (vol/vol) fetal bovine serum (Gibco BRL, Grand Island, NY, USA), 100 U/mL penicillin, and 100 µg/mL streptomycin. Cells were maintained in humidified atmosphere of 5% CO_2_ at 37°C. The culture medium was replaced with fresh medium every 2 to 3 days.

### Cell proliferation

Cell proliferation was determined by the Thiazolyl blue tetrazolium bromide (MTT) assay. D407 cells were seeded at a density of ten thousand per well in a 96-well plate and incubated with various concentrations of glucose at 37°C under 5% CO_2_ for 6 h–14 d. MTT was added into the media at the final concentration of 0.5 mg/ml for 4 h to allow MTT to be metabolized. After media were dumped off, cells were resuspended in formazan (MTT metabolic product) in 200 µl dimethyl sulfoxide (DMSO). Optical density was read at 540 nm and background was subtracted at 670 nm.

### Indirect determination of nitric oxide production

Nitric oxide (NO) production was determined by measuring extracellular nitrate/nitrite concentrations in culture supernatants. After pretreatment with different concentrations of S,S′-1,3-phenylene-bis(1,2-ethanediyl)-bis-isothiourea•2HBr (PBITU, 0.2, 0.4, and 0.8 mM, Santa Cruz Biotechnology, CA, USA), the cells were cultured in hyperglycemic conditions for 0, 6, 12, 24 and 48 h, and NO production was assessed with Griess reaction. Cell supernatants (1 000 µL) were mixed with 1 000 µL of the Griess reagent, a mixture (1∶1) of 0.2% naphthylethylene-diamine and 2% sulfonamide in 5% phosphoric acid. After 10-min incubation at room temperature, the absorbency was read at 540 nm on a spectrophotometer. The nitrate/nitrite levels were extrapolated from NaNO_2_ calibration curve.

### Quantitative real-time polymerase chain reaction (Q-PCR)

After pretreatment with different concentrations of PBITU ( 0.2, 0.4, and 0.8 mM) in the presence or absence of 1.0 mM L-arginine (Santa Cruz Biotechology), high glucose cultured D407 cells were collected to isolate total RNA using the Trizol RNA extraction reagent (Invitrogen, Guangzhou, China) according to the manufacturer's instructions. Purified RNA was reverse-transcribed using a SYBR PrimeScriptTM RT–PCR Kit (TaKaRa Corp, Dalian, China). Real-time quantification of human MDR1, iNOS, and pregnane X receptor (PXR) mRNA were performed on an ABI PRISM 7000 Sequence Detection System using SYBR Green I as the reporter dye (TaKaRa Corp). The comparative Ct method was employed while the relative quantity of the target gene mRNA, normalized to GAPDH and relative to the calibrator, was expressed as fold change = 2^−ΔΔCt^. The primers used for amplification were from different exons and their sequences were as follows: MDR1, 5′-TGGCACCCAGCACAATGAA-3′ and 5′-CTAAGTCATAGTCCGCCTAGAAGCA-3′; iNOS, 5′- GACTTCTGTGACCTCCA -3′ and 5′- GGTGATGCTCCCAGACAT -3′; PXR, 5′-GGCCACTGGCTATCACTTCAA-3′ and 5′-GTTTCATGGCCCTCCTGAAA-3′; GAPDH, 5′-GCA CCG TCA AGG CTG AGA AC-3′and 5′-TGG TGA AGA CGC CAG TGG A-3′. Duplicate PCR reactions were tested using the following amplification protocol: 95°C for 10 s followed by 30 cycles at 95°C for 5 s and at 60°C for 31 s.

### Western blot analysis

Proteins were separated using 12% SDS-polyacrylamide gels. The resolved proteins were transferred electrically to PVDF membranes and incubated with 5% skim milk in TBS with 0.05% Tween-20. The membranes were probed with primary antibodies for P-gp (C219, Calbiochem, CA, USA) and iNOS (iNOS antibody, Cell Signaling Technology, Shanghai, China) in blocking buffer overnight at 4°C. They were then incubated with horseradish peroxidase (HRP)-conjugated anti-mouse and anti-rabbit secondary antibodies (Santa Cruz Biotechnology) for 1 h at room temperature. As an internal control, the levels of ACTB (β-actin) were examined at the same time. After washing, the immunoreactive bands were detected using ECL chemiluminescence reagents. For P-gp expression, mouse fibroblast cell line NIH/3T3 (ATCC, CRL-1658) known for expressing low/no P-gp was used as a negative control [24]; and MDR cell line KB-V1 (Center of Experimental Animal, Sun Yat-sen University, Guangzhou, China), which expresses high levels of P-gp [25], was included as a positive control. The density of the bands was quantified using a laser densitometer (ATTO densitograph 4.0, Fujifilm, Tokyo, Japan). Each experiment was repeated at least three times.

### Rhodamine 123 accumulation assay

P-glycoprotein function was determined by rhodamine 123 (Invitrogen, Guangzhou, China) accumulation assay. Rhodamine 123, a fluorescent substrate for efflux transporters, has been used as a marker to study the function of P-gp in various MDR cells and various normal tissues including the human outer BRB [15], [23], [26]. Fluorescence intensity of intracellular rhodamine 123 was determined by flow cytometry. D407 cells were cultured in six-well plates at a density of 5×10^5^ cells/well and were loaded with rhodamine 123 (10 µg/mL) for 1 h at 37°C in the dark. After 1 h incubation, cells were washed and fed with rhodamine 123-free culture medium. To exclude dead cells, cells were then stained with 5 µg/mL propidium iodide (PI), a non-permeant dye that cannot stain living cells for 10 min. Cells were trypsinized, washed twice with cold PBS, resuspended in 200 µl PBS, and analyzed immediately by flow cytometry analysis using a BD FACS AriaTM flow cytometer and BD FACSDiVa software (Becton Dickinson, CA, USA). Photomultiplier settings were adjusted to detect green fluorescence of rhodamine 123 on the filter detector at an excitation wavelength of 488 nm and emission wavelength of 525 nm, and detect red fluorescence of PI at an emission wavelength of 620 nm. In each experiment, at least 20,000 events were analyzed. All experiments used six wells per condition and were repeated on two to three separate occasions. The mean fluorescence intensity in arbitrary units was used for data presentation.

To check the integrity of the RPE monolayer, the transepithelial electrical resistance (TEER), expressed as Ω•cm^2^, was measured with an epithelial voltmeter (MILLICELLERS; Millipore, Billerica, MA, USA) at the end of the rhodamine 123 accumulation experiments.

### Statistical analysis

All data were expressed as mean ± standard error (SE). Statistical significance was determined using Student's t-test (two-tailed) or one way ANOV followed by appreciated multiple post hoc testing. A probability (*P*) value of less than 0.05 was considered statistically significant.

## Results

### Decreased expression of P-gp at both mRNA and protein levels in the high glucose cultured RPE cells

**Figure 1:**
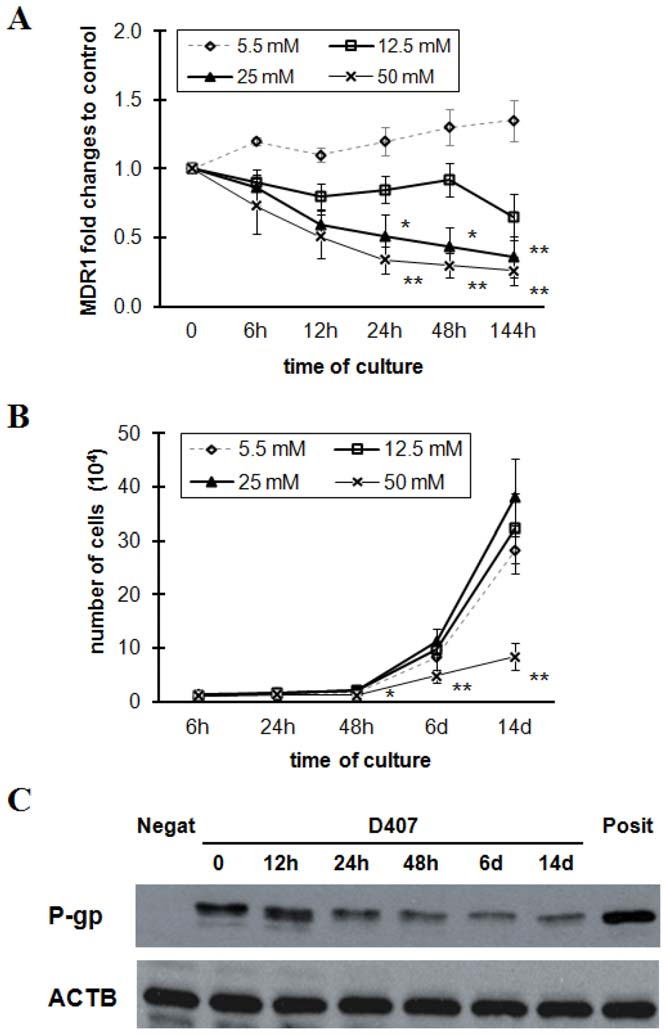
Expression changes of MDR1 gene and p-glycoprotein by exposure of high glucose. (A) D407 cells were incubated for 6–144 h in intermediate (12.5 mM) or high glucose (25 or 50 mM) medium and then MDR1 mRNA levels were assessed by quantitative real-time polymerase chain reaction. Results are normalized to control cells. Cells cultured in normal glucose were used as control. Data are means ± SE of four independent experiments. ^*^ *P*<0.05, **P<0.01 vs the normal controls. (B) RPE cells were exposed to 5.5, 12.5, 25, or 50 mM glucose for 6–144 h and then cell proliferation by Thiazolyl blue tetrazolium bromide (MTT) assay was determined. ^*^ *P*<0.05, **P<0.01 vs the controls. (C) D407 cells were treated with 25 mM for designed time periods. The expression of p-glycoprotein and ACTB were determined by western blots. ACTB was used as loading control. Mouse fibroblast NIH/3T3 cells and multidrug resistant human cervix carcinoma KB-V1 cells served as negative and positive controls, respectively. The data represent a western blot analysis from an individual experiment performed at least 3 times. P-gp, p-glycoprotein; Negat, negative control; Posit, positive control.

To examine the changes of P-gp expression across time at the outer BRB under hyperglycemic conditions, we first examined the MDR1 mRNA level in the RPE cells incubated for 6–144 h in intermediate (12.5 mM) or high glucose (25 or 50 mM). The incubation with 5.5 mM normal glucose was used as control. As show in Figure 1A, exposure to high, but not intermediate, glucose induced a significant decrease in MDR1 mRNA level by 24 h, when compared with normal control. This decrease did not recover to the normal level after 144 h incubation with high glucose. However, 50 mM glucose injured proliferation of the RPE cells after 48 h incubation (Figure 1B). Thus, RPE cells were exposed to 25 mM glucose in the subsequent experiments to manifest the high glucose effects. Consistently, the protein level of P-gp determined by western blot analysis was also decreased with high glucose treatment for 24–144 h (Figure 1C). This correlated well with the MDR1 mRNA level. In view of this, subsequent studies were performed majorly with 25 mM glucose incubation for 24 h. The down-regulation of P-gp was also observed following long-term (14 days) exposure to high glucose (Figure 1C, lane 7), a situation that mimics better the chronic hyperglycemia of diabetes.

### Elevated expression and activity of iNOS in the high glucose cultured RPE cells

**Figure 2:**
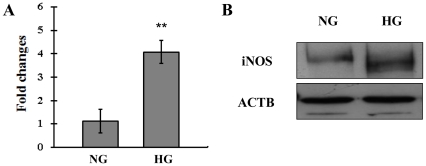
Expression levels of mRNA and protein of iNOS in the high glucose cultured human RPE cells. (A) Expression iNOS mRNA in D407 cells was determined by real-time polymerase chain reaction. The change in gene expression is expressed as fold change in relation to the control. Data are means ± SE of four independent experiments. **P<0.01 compared with cells incubated in the control. (B) Western blot analysis using monoclonal antibodies against iNOS and ACTB. The data represent a western blot analysis from an individual experiment performed at least 3 times. NG, normal glucose; HG, high glucose; iNOS, inducible nitrate oxide synthase.

**Figure 3:**
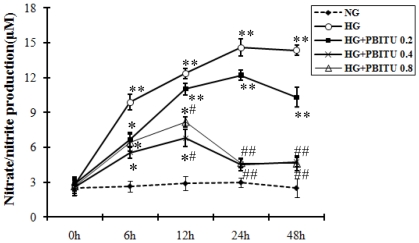
Indirect nitric oxide production by D407 cells following high glucose and iNOS inhibitor incubation. After 1 h preincubation with different concentrations of iNOS inhibitor PBITU, cells were cultured in high glucose for 0–48 h. Nitric oxide production was assessed by measuring extracellular nitrate/nitrite concentrations in culture supernatants using Griess reaction. Data are means ± SE of four independent experiments. *P<0.05, **P<0.01 compared with cells incubated in control; ^#^ *P*<0.05, ^##^ *P*<0.01 compared with cells incubated in high glucose. NG, nomal glucose; HG, high glucose; HG+PBITU 0.2, high glucose group pretreated with PBITU 0.2 mM; HG+PBITU 0.4, high glucose group pretreated with PBITU 0.4 mM; HG+PBITU 0.8, high glucose group pretreated with PBITU 0.8 mM.

Accumulated evidences have demonstrated that hyperglycemia induces iNOS gene expression and nitrosative stress [27], [28], [29]. To verify the role of iNOS induction at the outer BRB under hyperglycemic conditions, we firstly set to examine the iNOS expression by Q-PCR and western blot. As shown in Figure 2, high glucose induced significant increase in the expression at both the mRNA and protein levels after incubation for 24 h. It is known that iNOS produces large amounts of NO as a defense mechanism in various tissue. Then we assessed iNOS activity by measuring extracellular nitrate/nitrite concentrations as an indirect measure of iNOS-mediated intracellular NO production. In consistent with the upregulation in iNOS expression, cell supernatant nitrate/nitrite increased markedly by 6 h when compared with normal glucose medium, and reached a peak by 24 h after high glucose incubation (Figure 3). These findings indicate that high glucose activates the iNOS/NO induction in the cultured human RPE cells.

### Decreased P-gp expression at both mRNA and protein levels by high glucose involves iNOS

**Figure 4:**
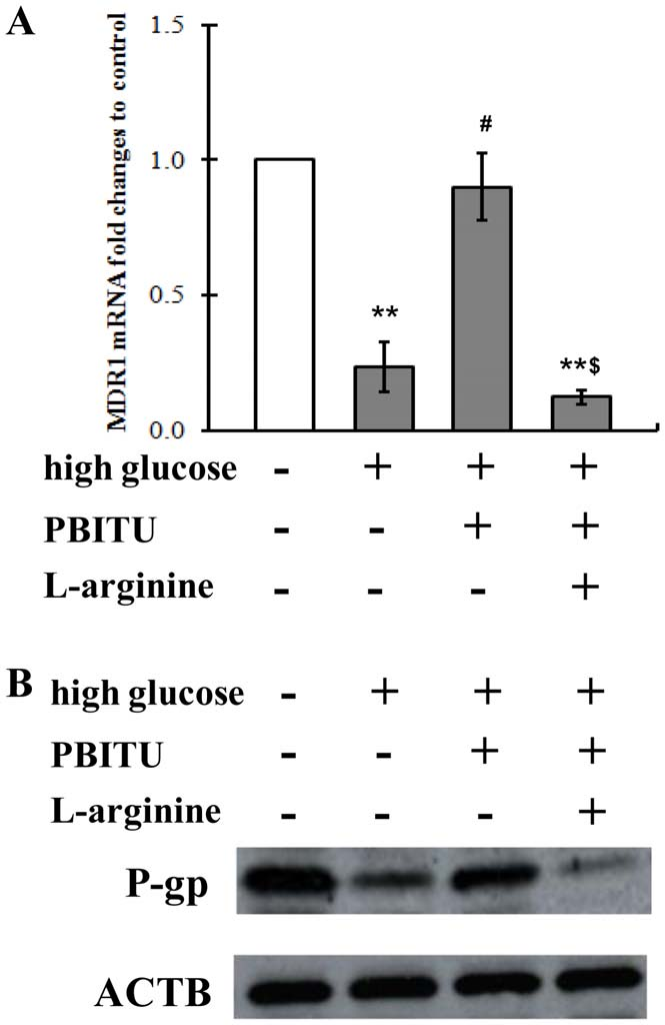
Decreased p-glycoprotein expression by high glucose involves iNOS. (A) Cells incubated in high glucose were pretreated with iNOS inhibitor PBITU (0.4 mM) alone or combined with nitric oxide donor L-arginine (1.0 mM) for 1 h. Expression of MDR1 mRNA in D407 cells was determined by quantitative real-time polymerase chain reaction. The change in gene expression is expressed as fold change in relation to the control. Data are means ± SE of four independent experiments. **P<0.01 compared with cells incubated in normal glucose medium; ^#^ *P*<0.05 compared with cells incubated in high glucose; ^$^ *P*<0.05 compared with cells incubated in high glucose and PBITU. (B) Western blot analysis using monoclonal antibodies against p-glycoprotein and ACTB. The expression of ACTB was used as a control. The data represent a western blot analysis from an individual experiment performed at least 3 times. P-gp, p-glycoprotein.

One technique commonly used to investigate the role of iNOS induction is administration of selective iNOS inhibitor such as PBITU. We first set a pilot study to determine the concentration of PBITU which abolished NO production by iNOS and found that the high glucose-stimulated NO production after 12 h was successfully blocked by 1 h preincubation of the RPE cells with 0.4 or 0.8 mM PBITU (Figure 3). Thus subsequent PBITU experiments were performed with a concentration of 0.4 mM. Then we set to determine whether iNOS was involved in the high glucose-decreased P-gp expression or not. As shown in Figure 4, the PBITU pretreatment significantly ameliorated the high glucose-decreased P-gp expression at the mRNA (Figure 4A, compare bars 2 and 3) and protein levels (Figure 4B, compare lanes 2 and 3).

Although the above results showed that high glucose stimulated iNOS induction and subsequent NO production (Figure 2 and 3), it was unclear whether NO could affect the P-gp expression. To test this, we next examined the effect of the NO donor, L-arginine, on P-gp alteration by high glucose. As shown in Figure 4, pretreatment with the addition of 1.0 mM L-arginine blocked the effects of PBITU on the P-gp expression at mRNA levels (Figure 4A, compare bars 3 and 4) and at protein levels (Figure 4B, compare lanes 3 and 4) in high glucose cultured D407 cells.

### Decreased P-gp activity by high glucose involves iNOS

**Figure 5:**
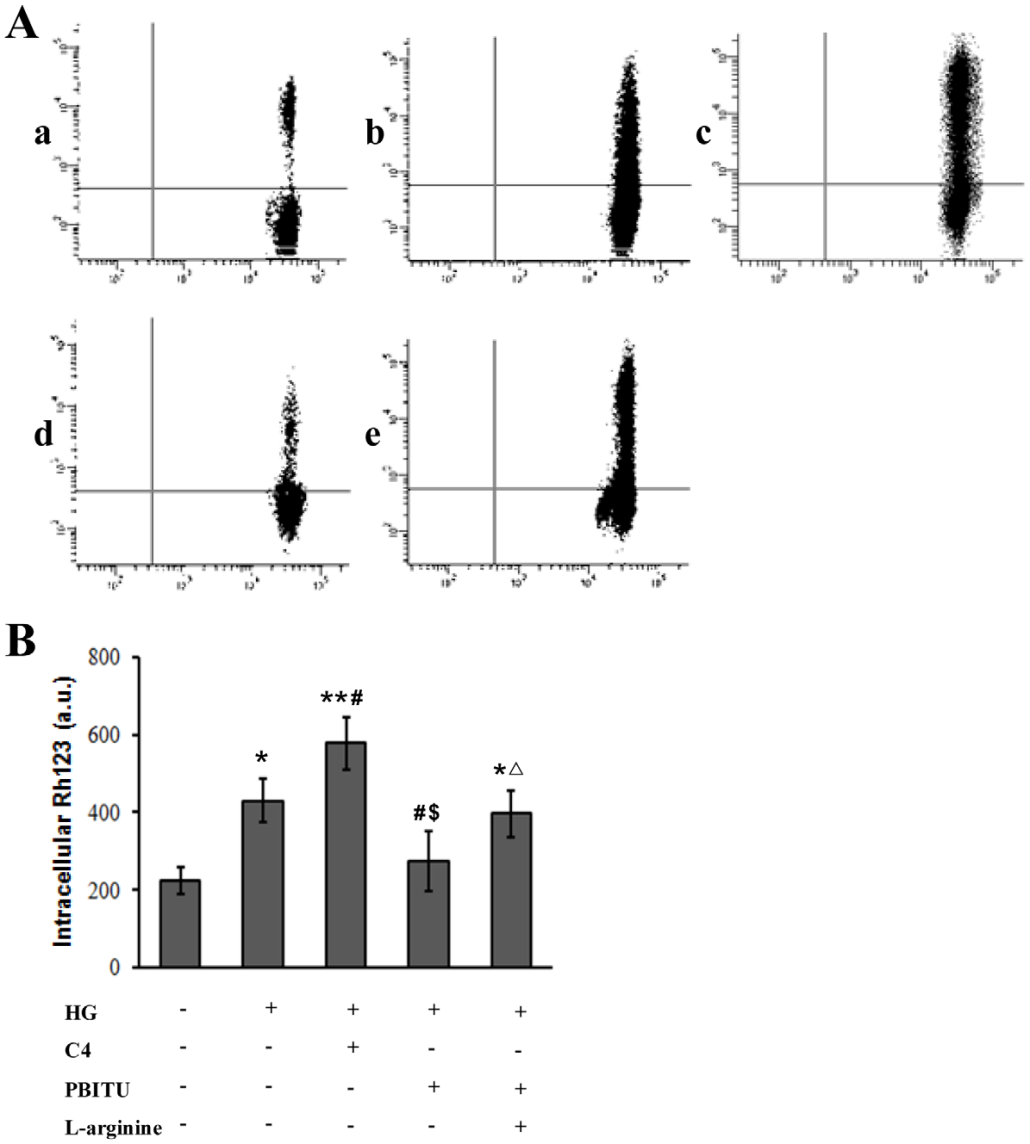
Decreased P-gp functional activity by high glucose involves iNOS. (A) P-gp function was determined by intracellular rhodamine 123 accumulation by flow cytometry. D407 cells were incubated in normal (a) and high glucose medium (b–e), respectively. Cells incubated in high glucose were pretreated with C-4 (c), or PBITU alone (d) or combined with L-arginine (e) for 1 h. Following the treatment, cells were then loaded with a rhodamine 123 (10 µg/mL). The mean fluorescence intensity of intracellular rhodamine 123 was determined by flow cytometry. The results shown are representative of those for three separate experiments. (B) The bar represents the mean ± SE for the three separate experiments. *P<0.05 and **P<0.01 compared with cells incubated in normal glucose medium; ^#^ *P*<0.05 compared with cells incubated in high glucose; ^$^ *P*<0.05 compared with cells incubated in high glucose and C4; ^Δ^ *P*<0.05 compared with cells incubated in high glucose and PBITU. HG, high glucose.

To assess the functional capacity of P-gp, cells were incubated with rhodamine 123 and intracellular rhodamine 123 concentration was measured by flow cytometry analysis. As shown in Figure 5, intracellular rhodamine 123 of D407 cells cultured in high glucose was significantly increased when compared with normal glucose controls, indicative of decreased P-gp function. C4, a cell-permeable cinnamoyl compound that reversibly inhibits P-gp efflux function with no noticeable effect on P-gp expression [30], [31], further augmented the high glucose-mediated intracellular accumulation of rhodamine 123. The high glucose-mediated intracellular accumulation of rhodamine 123 was reversed by the pretreatment with PBITU 0.4 mM for 1 h. These effects of PBITU were antagonized with the addition of 1.0 mM L-arginine. Collectively, these results suggest that functional expression of P-gp in the RPE cells under hyperglycemia involves iNOS induction.

### Expression of the nuclear receptors PXR mRNA by high glucose

**Figure 6:**
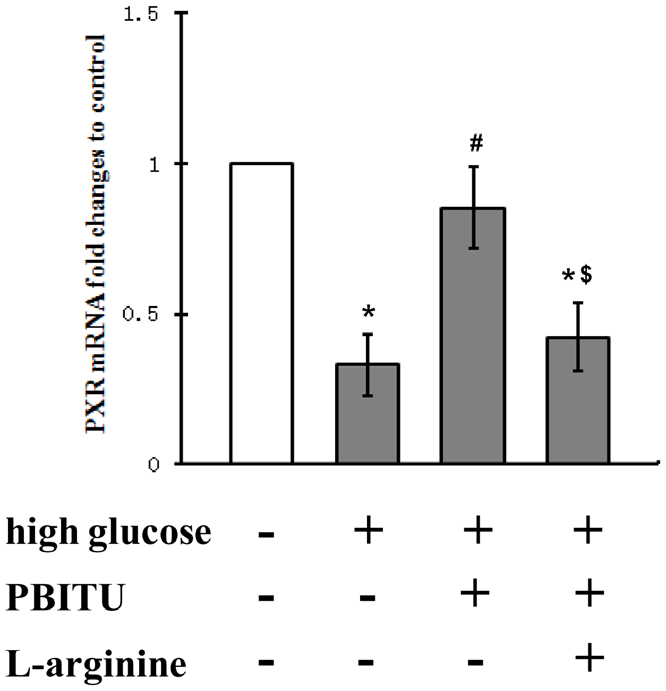
Expression levels of the nuclear receptors PXR mRNA in cultured RPE cells. Expression of PXR mRNA in D407 cells was assessed by real-time polymerase chain reaction. Cells incubated in high glucose were pretreated with PBITU (0.4 mM) alone or combined with L-arginine (1.0 mM) for 1 h. The change in gene expression is expressed as fold change in relation to the control (normal glucose). Data are means ± SE of four independent experiments. *P<0.05 compared with cells incubated in the control; ^#^ *P*<0.05 compared with cells incubated in high glucose; ^$^ *P*<0.05 compared with cells incubated in high glucose and PBITU.

Recent studies have revealed that PXR can function as a master regulator to control the expression of phase I and phase II drug-metabolizing enzymes, as well as members of the drug transporter family, including MDR1 [21], [32]. To further elucidate the iNOS pathway in cultured RPE cells after exposure of high glucose, we next assessed the expression level of this nuclear receptor. As shown in Figure 6, Q-PCR revealed that the expression of PXR after high glucose incubation was lower than basal level. However, the pretreatment of PBITU partially blocked the downregulation of PXR mRNA by high glucose. Furthermore, the PBITU blockade was antagonized with the addition of L-arginine. These results suggest that downregulation of PXR by high glucose is mediated at least partially through the iNOS induction.

## Discussion

The retinal pigment epithelium plays a central role in retinal physiology by forming the outer BRB and supporting the function of the photoreceptors. Alterations of this barrier have been associated with various sight-affecting conditions, including DR [6], [8], [30]. New therapeutic strategies addressed to modulate RPE impairment are therefore warranted. Recently, changes of P-gp, a well-known efflux pump, have been implicated in the pathogenesis of many tissues under diabetic conditions [16], [17], [18], [19], [20]. In the present study, we first asked whether the P-gp at the human outer BRB would be affected similarly. It is becoming increasingly clear that iNOS activity is induced in DR [27], [28], [29], and that NO is a crucial regulatory mediator for functional expression of the barrier transporters [33], [34], [35]. Thus, whether the iNOS induction was involved in the regulation of P-gp at the outer BRB or not would be our next question.

Our first key finding demonstrated that the basal levels of transcriptional, translational, and functional P-gp in cultured human RPE cells could be inhibited under hyperglycemic conditions. This finding has important clinical implications. According to the documented studies, a function of P-gp to RPE is to limit the accumulation of toxic drugs or metabolites from the subretinal space and thus serves a protective role for the neural retina. Inhibition of P-gp by high glucose will potentially decrease the normal transport function of the RPE. Another characteristic of P-gp is to expel a broad range of hydrophobic compounds including certain hydrophobic drugs widely used in clinical therapy, such as steroids. From this view, we can also postulate that decreased P-gp expression at the outer BRB may increase the penetration of therapeutic agents targeted to specific areas of the retinal neuron. Mechanisms responsible for altered P-gp in this study have not been extensively investigated, but subsequent deterioration of the outer BRB and alteration of pharmacokinetics of therapeutic drugs are certainly possibilities. They imply increased difficulties in protection against neurotoxicants and alterations in pharmacokinetics of therapeutic drugs targeted to the retinopathies.

Since diabetes is known to produce a nitrosative stress condition and the exposure of NO may lead to a regulation of the gene expression of efflux transporters [34], [35], the role of NO in the functional alterations of P-gp at the BRB was of particular interest. Here we found that the decreased expression and activity of P-gp was associated with an iNOS induction and subsequent NO production in the high glucose cultured RPE cells. Moreover, the pretreatment with PBITU partially blocked the decreased functional expression of P-gp. The addition of L-arginine antagonized the blockade effects of PBITU. It is because that PBITU competes with L-arginine for the binding site on iNOS [36]. Hence, pretreatment with PBITU only partially blocked iNOS activity and subsequent NO production. Our finding is consistent with previous investigation in experimental diabetes that iNOS induction is involved in the reduction of ileal P-gp expression. However, it does not agree with the finding from Maeng and colleagues [17]. They reported that nitrosative stress leads to up-regulation of P-gp at the blood-brain barrier of diabetic rats. The possible explanation for this discrepancy is that P-gp in different tissue barriers may have tissue-specific characteristic responses to the nitrosative stress, such as diabetes. Indeed, in another nitrosative stress model using lipopolysaccharide treatment, it has been demonstrated that expression and/or function of ABC transporters (i.e., P-gp) were differentially regulated in a tissue-specific manner. Namely, lipopolysaccharide-induced acute inflammation caused down-regulation of P-gp in the brain, the intestine, and the liver, but up-regulation of P-gp in the kidney [34], [37], [38], [39]. Since the findings from others and ours indicate the existence of functional efflux transporters including P-gp in different tissue barriers under diabetic conditions, a cell or tissue-specific regulatory mechanism of nitrosative stress may exist in the case of P-gp.

Nitric oxide is produced by different isoforms of NOS. In the retina, constitutive NOS and iNOS are present, the former in amacrine and ganglion cells and the latter in RPE and Müller cells [40], [41]. RPE cells from bovine [42], human [43], and murine [40] species have been identified containing the iNOS isoform. RPE cells have also been shown to produce NO in response to a number of cytokines [43], and it has been suggested that RPE-derived NO may be involved in the maintenance of barrier integrity and function [42], [44]. The present study suggests that iNOS/NO signaling pathway also has a role in the regulation of transporter functions at the human outer BRB. How the iNOS pathway and P-gp interact or coordinate at the outer BRB to the response to hyperglycemia is uncertain. However, it is possible that there is both some redundancy in the response, as well as cell and tissue-specific aspects to iNOS mediation. In the context of the present study, it is worth pointing out that iNOS has been added to the list of therapeutic targets for the DR. The upregulation of iNOS has been reported in retinas of experimental diabetic rodents and patients in most studies [27], [28], [29]. Recently, Zheng et al. [45] has demonstrated directly that the critic role of iNOS in the early stages of DR using mice genetically deficient in iNOS. Aminoguanidine, a relatively selective inhibitor of iNOS [46], has been found to inhibit the diabetes-induced iNOS expression and NO production in retina [47], and to subsequently inhibit the development of the DR [48]. Our finding of the restored functional expression of P-gp suggests that iNOS inhibitor has a beneficial effect on the integrity of the outer BRB under hyperglycemic conditions, and further supports the concept of inhibiting iNOS pathway as a therapeutic strategy for DR.

There are a number of transacting factors which are essential for the activation of human MDR1 gene at transcriptional level. Among them are expression and regulation of transcriptional factors such as Y-box binding-1 [49], Hypoxia-inducible factor 1 and PXR (also known as SXR in human) [35]. PXR is a member of a superfamily of ligand-activated transcription factors, the so-called orphan nuclear receptors. In hepatocytes, ligand-activated, nuclear receptors are transcriptional regulators of drug metabolizing enzymes and drug export pumps, but only one, the PXR, regulates P-gp expression. In the present study, the decreased mRNA levels of PXR, which were consistent with the attenuated expression of P-gp after incubation with high glucose (see Figure 1 and 6), indicate that PXR is a potential therapeutic target at the transcriptional level for the transport function at BRB under hyperglycemic conditions. To the best of our knowledge, this is the first evidence for PXR expression in the human RPE cells and for possible regulation by nuclear receptors of xenobiotic efflux pumps at the outer BRB. We then assumed that NO might be able to suppress PXR, leading to an inhibition of MDR1 and other PXR target genes. Although a previous report indicated that the promoter of iNOS contained a PXR-responsive element, and that increased expression of iNOS was a direct response to PXR activation [50], our results suggest that it is the iNOS induction which mediates PXR-associated events in the RPE cells. Consistent with this view is the finding from Mitchell et al. study that iNOS mediates contractility through PXR in uterine tissues [51]. Mechanisms responsible for this have not been identified, but tonic regulation of PXR by iNOS or by endogenous ligands produced under hyperglycemic conditions are certainly possibilities. In fact, PXR, other nuclear receptors, e.g., CAR, xenobiotic metabolizing enzymes, and efflux transporters have been demonstrated to comprise a regulated network of core defense mechanisms in the liver [52], [53]. Further investigation into the role of cell signaling pathways in iNOS-mediated transcription, and into signaling pathway crosstalk will be necessary to fully understand the functional implication of these signaling events at the human outer BRB.

In conclusion, our results provide clear evidence that iNOS induction participates the decreased expression and activity of P-gp by high glucose in the cultured human RPE cells, and indicate that iNOS signaling pathway has a novel role in the regulation of transporter expressions and functions at the human outer BRB. Taken in the context of previous studies demonstrating a role of iNOS activity, the concept of inhibiting iNOS pathway as a therapeutic strategy deserves further evaluation for the prevention of DR.
